# Determinants of fast attack performance on match outcome in the elite women’s ice hockey

**DOI:** 10.1371/journal.pone.0306469

**Published:** 2024-07-05

**Authors:** Naiyuan Tian, Gang Xu

**Affiliations:** 1 School of Physical Education, Yanshan University, Qinhuangdao, China; 2 College of Competitive Sports, Beijing Sport University, Beijing, China; Sunway University, MALAYSIA

## Abstract

The primary objective of this study was to discern the determinants affecting the ice hockey game based on the performance of the fast attack between the winning and losing teams. Data from the women’s ice hockey games at the Beijing 2022 Winter Olympics (n = 28) were used. A total of 2011 fast attacks were recorded, which included winning team 1156 times and losing team 855 times. 29 variables from nine categories were involved and analysed using chi-square tests, univariate tests and binary logistic regression. As a result, that fast attack performance varies between winning and losing teams, Effective Offensive Play. Scoring Analysis of the 2005 World Championships and the 2006 Olympics, INT, DZ, OZ, one-timer, dekes, shooting zone 1, shooting zone 3, shooting zone 4, SOG, SG%, 2^nd^ period, PK, are key variables in distinguishing the winner and loser (*P*<0.05). The predictive model shows that shooting zone 4 (OR = 0.824), one-timer (OR = 1.255), 2^nd^ period (OR = 1.193), SOG (OR = 1.230), and SG% (OR = 1.170) are determining factors of game outcomes. The current information has important practical applications as it allows coaches and players to improve the tactical strategy and offensive efficiency of the game.

## 1. Introduction

Ice hockey is a skill-dominated confrontational sport [1] which represents as one of the fastest sports around the world, and “faster” has always been the key point for the development trend of ice hockey. As we can conclude speed should be considered as the paramount feature for the ice hockey [2–5]. This era in particular, the development of world ice hockey, the upgrading of equipment, and the evolution of competitive patterns in sports itself determined that the speed of ice hockey games will continue to accelerate. Then the superiority of fast attack tactics in the game is increasingly prominent.

Fast attack is an important part of the tactical system of team sports, although the terms of fast attack are different between various sports (e.g. basketball and handball called it as fast break, football and water polo generally define it as counter-attack, etc.; [6–8]), however, their general character is alike. The purpose is to advance as fast as possible to the opponent’s goal at the moment of transition from defence to offence, strive to create a numerical advantage, and finally score a goal [9–12]. Many studies on match analysis have found that the performance of the team’s fast attack tactics has a significant influence on the match outcome (i.e. win or lose; [12–15]). According to the International Ice Hockey Federation (IIHF) coaching manual published in 2007, a counter-attack can either be done by fast attack quickly or with a mode of puck control. Johnston & Walter [1] pointed out in their work that counter-attack often leads to more odd-man rush situations during the game due to the offensive side will generally attack with all its strength, and when losing possession, it usually cannot return to the defensive position immediately right after the transition. In 2008, the International Ice Hockey Centre of Excellence (IIHCE) reported that in the top Division, the scoring rate of fast counter-attacks in the tournament is 14%, which is considerably higher than the organised attack with a scoring rate of 1.6%. Based on the above studies, it is obvious that the fast attack is an irreplaceable part of the game and is an effective offensive tactic to score goals.

So far, most studies on the game performance of ice hockey are regarding technical movements and physical tests such as skating, off-ice fitness, repeated sprint ability [16–18] and shooting [19], etc. Nevertheless, analyses of tactical performance are scarce. To our knowledge, Barilla et al. [20] and Liitola [21] have respectively conducted targeted research on power play, one-on-one battles and face-off. However, there are limited research has been found regarding fast attack tactic, which generally considered a highly utilized tactic in hockey games. Given the rationale stated, the main purpose of the current study was to discern the determinants that affect the ice hockey match according to the fast attack performance between winners and losers. We screened 29 indicators [22], including nine dimensions which are ways to gain possession, zone of gaining possession, shot type, shooting zone, shooting performance, game period, strength, scoring chance, and outcome of fast attack. This data could provide coaches and players with optimal tactical strategies in the game, thus improving the team’s overall offensive effectiveness.

## 2. Methods

### 2.1 Sample

The sample for this study comprised all 28 matches (i.e. 20 group matches and 8 playoffs) of the 2022 Winter Olympic Games women’s ice hockey that took place in Beijing (China) from 3 to 17 February 2022. The games involving 10 national teams were those: Canada, U.S.A, Finland, Switzerland, Russia, Japan, the Czech Republic, Sweden, China, and Denmark.

A total of 2011 fast attacks were video-recorded and analysed with a domestic laptop computer (Lenovo Ryzen 5-5500U CPU) operating Microsoft Windows 10 (Microsoft® Corp, U.S.A.) to achieve the purpose of the study. Those fast attack indicators with extremely small amounts of data were excluded (n = 71; 3.5%). The video data of these matches were collected from public-accessed websites (https://v.qq.com/), which is the resource from the live broadcast of the game. And the few routine statistics required for the study are gained from an official website of the IIHF (https://www.iihf.com/). All data related to fast attack performances were collected by the same observer (more than fifteen years of professional player experience) for the video observing. The information obtained from the video is gathered by Microsoft Excel 2019 (Microsoft® Corp, U.S.A.). Considering the reliability of the sample, we only select the results of similar teams and exclude the teams with large differences, because when the difference between the two sides is too large, it may not only affect the final outcome of the game, so these games need to be removed from it.

### 2.2 Variables

The spreadsheet recording the fast attack variables was divided in two groups, those from winning teams (n = 28) and losing teams (n = 28). Afterwards, the variables were organised into nine main dimensions, including 29 independent variables (see Table 1) concerning fast attack performance which were selected based on previous researches [23–25], and with repeated revisions, it has been approved by 20 experts, consisting of 16 national team players with a minimum 15 years of experience and four senior coaches recognised by the Chinese Ice Hockey Association (CIHA).

**Table 1:** Definitions and descriptions of the categories and variables analysed in the study.

Categories	Variables	Definition & description
Ways of gaining possession	TkA	The player takes the puck from the opposition, rather than gaining possession through an opposition error.
	INT	Stopping the pass of the opponent.
	TO	Lost puck due to personal error.
	Face-off	Puck is gained by a successful face-off.
	Rebound	Puck is gained by a rebound created by an opponent’s shot.
Zone of gaining possession	DZ	Puck is gained in DZ.
	NZ	Puck is gained in NZ.
	OZ	Puck is gained in OZ.
Shot type	Wrist shot	Shot released by a rotational wrist action (the puck doesn’t separate from blade of stick).
	Snap shot	Shoot with a quick delivery of the puck into the goal.
	Slap shot	A hard shot which takes a big swing before hitting the puck with the blade of the stick.
	One-timer	Shot to the goal without stopping the puck.
	Dekes	A shot made by a fake move to deceive a goaltender.
Shooting zone (see Fig 2)	Zone 1	Shooting in 1 zone.
	Zone 2	Shooting in 2 zone.
	Zone 3	Shooting in 3 zone.
	Zone 4	Shooting in 4 zone.
	Zone 5	Neutral in 5 zone.
Shooting performance	SOG	Shot on goal in a fast attack.
	SG%	Fast attack shot percentage.
Game period	1^st^ period	The fast attack in the 1^st^ period.
	2^nd^ period	The fast attack in the 2^nd^ period.
	3^rd^ period	The fast attack in the 3^rd^ period.
Strength	Equal	Fast attack with an equal number of players.
	PP	Fast attack with a superior number of players.
	PK	Fast attack with a numerical disadvantage of players.
Scoring chance	Odd-man rush	The offensive side outnumbers the defensive side when they are entering the attacking zone (only covers 1–0, 2–1, 3–2).
Outcome	Successful	Considered as goals, shots on goal, creating a rebound after a shot, and creating a penalty on the opponent.
	Unsuccessful	Considered as a shot missed the goal, the opponent steals the puck away, errors of handling or passing, and offside.

Note: TkA: Takeaway; INT: Interception; TO: Turnover; DZ: Defensive zone; NZ: Neutral zone; OZ: Offensive zone; SOG: Shot on goal; SG%: Shot percentage; PP: Power play; PK: Penalty kill.

As an assessment for intra-observer reliability of the observational analysis made, 2 games were randomly selected and recorded twice through a test-retest viewing within a minimal 4-weeks interval.

### 2.3 Statistical analysis

In the course of statistical validation, a crossover analysis was conducted using chi-square (x^2^) to identify the difference in fast attack outcomes from winners to losers. Then, the assumption of normality for the statistics was performed with the Shapiro-Wilk test to examine whether parametric or non-parametric analysis would be used. Following this, paired sample t-test (i.e. parametric) [23] and Wilcoxon signed rank test (i.e. non-parametric) were considered to check for univariate difference among winners and losers teams. Subsequently, it was constructed with a binary logistic regression to predict the outcome of the match based on a set of independent variables for both groups [24], the forward stepwise method was used. The dependent variables to be used within the model would be Y = 0, 1,…, where 0 indicates a loss and 1 indicates a win. The logistic regression equation is as follows:

$$P(y=1)=e^{z}/1+e^{z}$$ (1)

$$Z=B_{0}+B_{1}X_{1}+B_{2}X_{2}+\ldots +B_{m}X_{m}$$ (2)

Where *P* represents the probability of occurrence under the influence of multiple independent variables *X* and *B*_*0*_ represents the constant coefficient.

All data were analysed with the statistical package SPSS 26.0 (IBM® Corp, U.S.A.). Descriptive statistics were presented as mean ± SD. In addition, Cohen effect sizes (ES) were calculated to provide a more meaningful result. ES = 0.2–0.5 was considered small, 0.5–0.8 was moderate, and >0.8 was a large effect size [25,26]. The significance level was set at *P*<0.05.

## 3. Results

As a result, there were a total of 2011 (36.0±10.0) fast attacks during the women’s ice hockey competition at the Beijing 2022 Winter Olympics, of which 1050 (18.0±7.1) successful fast attacks (116 goals) and 961 (17.2±4.4) unsuccessful fast attacks, accounting for 52.2% and 47.8%. The winners had 1156 (41.3±1.8) attempts, including 637 (22.8±6.0, 31.7%) successful fast attacks (91 goals) and 519 (14.8±5.9, 25.8%) unsuccessful fast attacks, while the losers had 855 (30.8±1.3) attempts, consisting of 413 (18.6±4.8, 20.5%) successful fast attacks (25 goals) and 442 (15.8±3.5, 22.0%) failed, with a chi-square value (x^2^) of 9.108^a^, *P* = 0.003, indicating a significant difference (*P*<0.01) in fast attack outcomes between winners and losers.

The results given by the univariate analysis (see Table 2) revealed significant differences in 14 indicators (*P*<0.05), which were TkA, INT, DZ, OZ, one-timer, dekes, shooting zone 1, shooting zone 3, shooting zone 4, SOG, SG%, 2^nd^ period, PK. Among which INT (ES = 0.885), DZ (ES = 0.815), one-timer (ES = 0.969), shooting zone 1 (ES = 0.913), SOG (ES = 0.906) between winners and losers were found with larger effect sizes (ES>0.8). The winners outperformed the losers in the mean values of most significant variables, the losers were higher than the winners only in the application rates of INT, DZ, and shooting zone 4, which to some extent suggest that these indicators are characteristics of the weak team’s fast attack, while the winner impressed in steals (TkA), shooting skills (one-timer), and shooting performance, but surprisingly no significant differences found in scoring chances between the two groups.

**Table 2:** Description statistics and univariate differences in fast attack variables.

Categories	Variables	Winners (n = 1156)			Losers (n = 855)			*P*-value	ES
		M	SD	%	M	SD	%		
Ways of gaining possession	TkA	21.2	4.7	51.3	13.7	6.1	44.9	0.002 ^a^**	0.727
	INT	4.6	2.7	11.1	5.1	2.5	16.7	0.002 ^a^**	0.885
	TO	7.2	3.8	17.4	5.9	3.1	19.3	0.225	0.269
	Face-off	6.5	3.7	15.7	4.1	2.6	13.4	0.418	0.228
	Rebound	1.7	1.1	4.1	1.9	1.5	6.2	0.051	0.515
Zone of gaining possession	DZ	14.7	4.4	35.6	13.0	2.9	42.6	0.011 ^a^*	0.815
	NZ	7.8	3.4	18.9	5.5	2.7	18.0	0.123	0.433
	OZ	18.8	6.9	45.5	12.0	3.9	39.3	0.040 ^a^*	0.619
Shot type	Wrist shot	6.4	3.4	15.5	5.3	2.2	17.4	0.411	0.205
	Snap shot	12.8	4.9	31.0	10.2	3.9	33.4	0.368	0.230
	Slap shot	1.7	1.9	4.1	1.4	1.2	4.6	0.443	0.164
	One-timer	7.0	3.6	17.0	3.4	1.9	11.1	0.002 ^a^**	0.969
	Dekes	2.1	1.5	5.1	1.0	1.4	3.3	0.022 ^b^*	0.539
Shooting zone	Shooting zone 1	11.8	5.3	28.6	6.5	3.5	21.3	0.002 ^a^**	0.913
	Shooting zone 2	4.5	2.6	10.9	3.5	2.3	11.5	0.584	0.152
	Shooting zone 3	3.9	2.3	9.4	2.1	1.4	6.9	0.014 ^a^*	0.626
	Shooting zone 4	3.0	1.9	7.3	3.6	2.2	11.8	0.005 ^a^**	0.667
	Shooting zone 5	8.6	3.5	20.8	6.5	2.7	21.3	0.959	0.014
Shooting performance	SOG	21.5	6.5	52.1	13.3	5.4	43.6	0.003 ^a^**	0.906
	SG%	14.7	8.0	-	8.1	10.2	-	0.004 ^b^**	0.714
Game period	1^st^ period	13.4	4.0	32.5	10.6	4.4	34.7	0.527	0.180
	2^nd^ period	15.4	3.6	37.3	10.0	2.8	32.7	0.040 ^a^*	0.629
	3^rd^ period	12.4	4.5	30.0	10.0	3.3	32.7	0.147	0.389
Strength	Equal	36.3	9.1	87.9	26.7	6.8	87.4	0.555	0.150
	PP	2.9	1.9	7.0	3.1	2.2	10.2	0.087	0.539
	PK	1.9	1.6	4.6	0.9	1.8	2.9	0.030^b^*	0.440
Scoring chance	Odd-man rush	2.3	1.3	5.6	1.4	1.4	4.6	0.145	0.309

Note: Signs indicate significantly different between both groups, using either the paired samples t-test (a) or the Wilcoxon signed rank test (b); *P<0.05; **P<0.01.

TkA: Takeaway; INT: Interception; TO: Turnover; DZ: Defensive zone; NZ: Neutral; OZ: Offensive zone; SOG: Shot on goal; SG%: Shot percentage; PP: Power play; PK: Penalty kill.

In Table 3 the six most appropriate variables for predicting the outcome of a match were displayed, four of them associated with the shot, illustrating the unparalleled importance of the shot to the success of the game. The results of the model show that Shooting Zone (shooting zone 4), Shot type (one-timer), Game period (2^nd^ period), Shooting Performance (SOG, SG%) are the key aspects of fast attack performance that can significantly influence the match outcome (*P*<0.05). With the negative beta of shooting zone 4 (*β* = -0.193, OR = 0.824), it is clear that for each additional shot in shooting zone 4, the ratio of match winning decreases by approximately 28%. While increasing the one-timer (OR = 1.255, each increase will improve the odds of winning by 25.5%), 2^nd^ period (OR = 1.193, each fast attack increase in the 2^nd^ period will improve the odds of winning by 19.3%), SOG (OR = 1.230, each increase will improve the odds of winning by 23.0%) and SG% (OR = 1.170, each additional unit increases the odds of winning by 17.0%) contribute positively to the success of the game, and the calculated probabilities are very close, with an average probability equal to 54.8%.

**Table 3:** Binary logistic regression predicting to match outcomes (winning and losing).

Variables	β	S.E.	*P*-value	OR	IC 95% OR		Probability
			(Wald)		Lower	Upper	
INT	-0.175	0.094	0.063	0.839	0.698	1.010	0.42
Shooting zone 4	-0.193	0.089	0.029*	0.824	0.693	0.980	0.41
One-timer	0.227	0.093	0.015*	1.255	1.045	1.507	0.56
2^nd^ period	0.177	0.080	0.027*	1.193	1.020	1.396	0.54
SOG	0.207	0.071	0.004**	1.230	1.069	1.414	0.55
SG%	0.157	0.069	0.023*	1.170	1.022	1.339	0.54
Constant	-16.721	6.122	0.006**				

Note: *β*: Regression coefficient, S.E: Standard error; *P*-value (wald): Significance of parameters with the Wald test, OR: Odds ratio, Cl: Confidence interval, Probability: Probability of winning; *P<0.05, **P<0.01.

**Fig 1:**
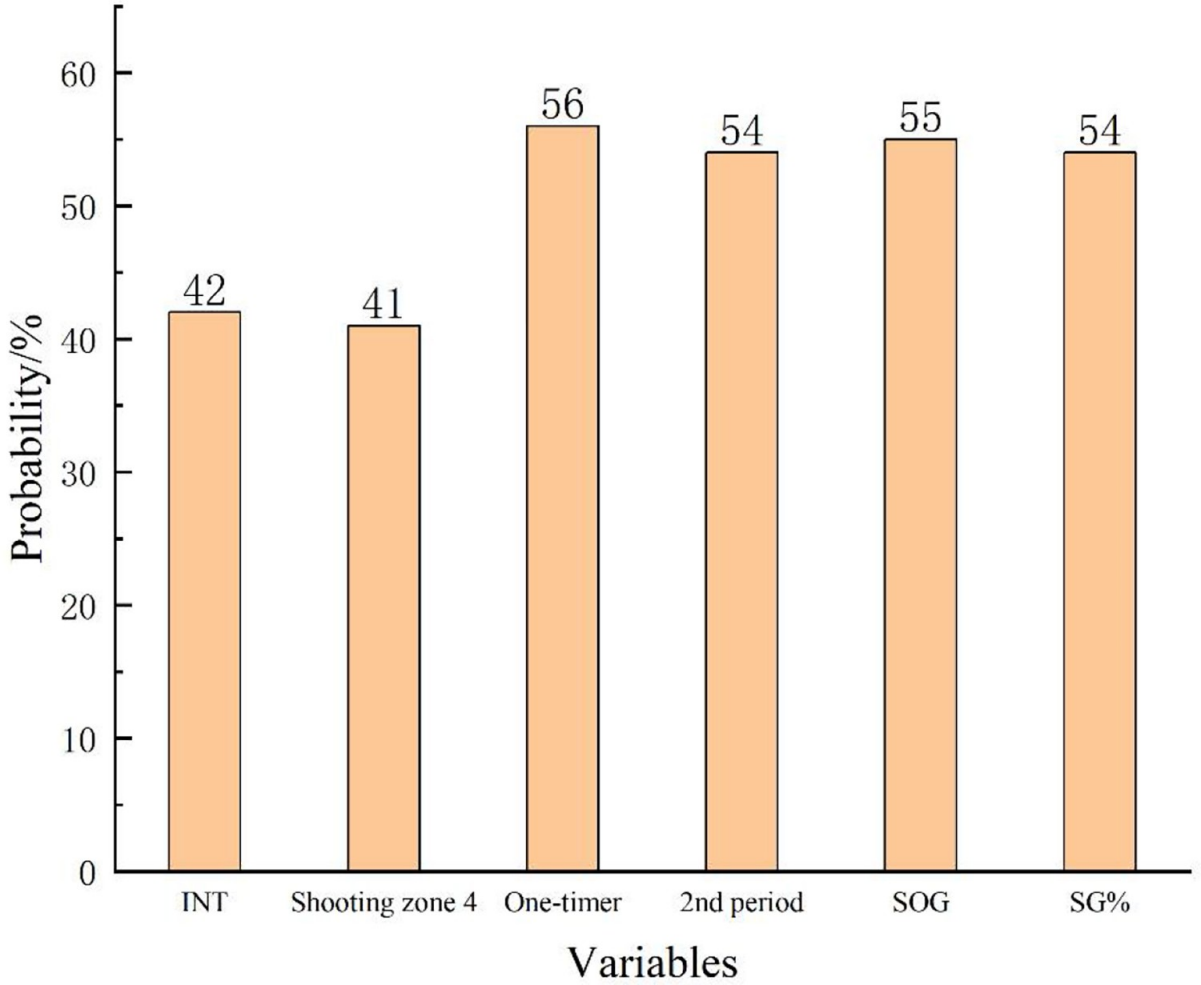
Probability of result.

According to the results of Fig 1, one-timer is the most important influencing factor, with a probability of 56%, followed by 2nd period, SOG, and SG%. These factors are quite important in winning international women’s hockey tournaments.

Based on the regression coefficients *β* presented in the model, the following logistic regression equation can be established:

$$\begin{array}{l} Z=-16.721-0.175(INT)-0.193(Shooting\;zone\;4)+0.227(One-timer)\\ +0.177(2^{nd}\;period)+0.207(SOG)+0.157(SG\%) \end{array}$$ (3)

The model was given an R^2^ Nagelkerke = 0.763, which represents a large effect size (>0.26, [27–30]). The Hosmer-Lemeshow test reveals that the model is fitting extremely well (*P* = 0.970, *P*>0.05), and overall accuracy of 89.3%% reported by the regression equation, which demonstrates that the model validity is reliable and has a great discriminant capacity.

The important factors of the game are shown in Table 4.

**Table 4:** The important factors of the game.

Influencing factors	Importance
Shooting zone	+++
Shooting type	++
Game period	++
Shooting performance	+++

## 4. Discussion and analysis

### 4.1 Discussion

Very little scholarly attention has been paid to ice hockey tactics as we mentioned above, especially when it comes to female ice hockey. This is the first targeted investigation on fast attack performance of elite female ice hockey players as such it provides new information on fast attack performance that distinguishes between winning and losing teams.

The fast attack is achieved by a transition of defence to offence, which means that the fast attack begins when the puck is regained. This suggests to us that if the two sides of the match are closer, the shorter the sustained possession of the puck in a single possession, the more frequent the transition pace between offence and defence, and the more fast attacks are likely to be made. On the basis of the results from the univariate analysis in this study, TkA and INT had a more meaningful impact on the outcome of the game than face-off, TO and rebound, it make a significant difference among the winners and the losers. The performance of TkA relied more on the ability of body checking, which is understandable given the increasing research on improving the special strength of ice hockey players. It should be clear that the effectiveness of body checking of the team in a match plays an important role in the coordination between the defensive players, when one defender battles with the puck carrier, the other one should be quick to take the puck away. This was noted which is a widely used way of gaining possession often seen in the games nowadays. Especially in the OZ, The results show that the winning team had more TkA and therefore initiated more fast attacks in the OZ, suggesting that better steals provide more fast attack opportunities in the OZ. This is usually achieved thanks to the team’s better forechecking, which refers to the act of checking your opponent in their defensive zone to regain the puck and continue your attack. This enables greater pressure to be applied during opponents break out, and this requires the players there to execute well-articulated forecheck and intensive, positive skating to make it happen. On the other hand, in terms of the INT, we learned that the losing team launched more fast attacks initiated by INT than the winning team, but in the regression model INT was not the determining factor of the game, as the weaker team had fewer opportunities to control the puck and therefore could only gain possession more by INT and TO and rebound, so the losing team launched more fast attacks from the DZ. The high ES of INT (ES = 0.885) shows that it is necessary to block the opponent’s passing routes, while the attackers without the puck are the key to increase the efficiency of the INT, because a successful passing depends not only on the concealment of the passing player, but also on the receiver’s timing.

Furthermore, where existing research has shown that 40.5% of the goals scored in the 2005 World Championships and 2006 Olympics were scored via one-timer, followed by Garbe’s study [31] of the National Hockey League goals in the 2006–2007 season, which found that top rank teams (top 10) accounted for 52.7% of all goals scored with one-timer shots. This is compared to previous studies [32] of 28% of one-timers in women’s ice hockey at the PyeongChang Winter Olympics, suggesting that there is a difference between men’s and women’s ice hockey differ in their use of shooting techniques, with men’s faster paced games requiring a greater need to minimise shot preparation time. This informs that one-timer is a key skill, and the ability and frequency of one-timer use is a decisive factor in winning a match. However, being fast is not enough, we also found that the winning teams were able to use more individual handling techniques in their shots on the fast attack and were better at making fake moves to add more difficulties for the goalkeeper. Of course, the conditions for the formation of such shots contain limitations, as the shot can only be taken when the puck carrier is facing the goalie, so it is more driven by the individual skills of the players, which also shows that the players of the winning team generally perform better in individual plays, depending mainly on their ability to read and react to different situations of the game.

**Fig 2:**
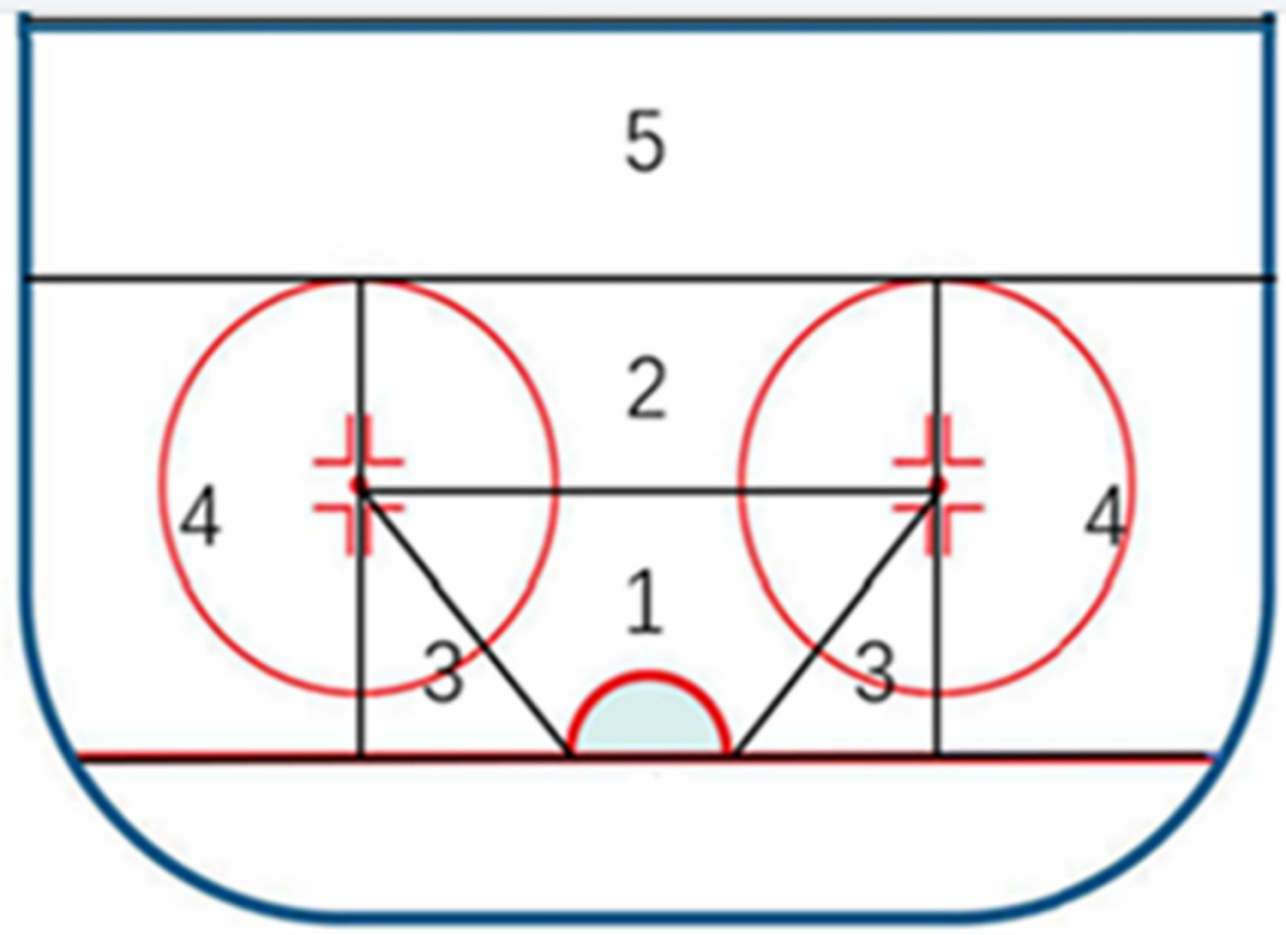
Distribution of shot zones.

It is well known that there is a strong correlation between the area of shooting and scoring, and the results of this study show that the winning team shoots significantly more in zones 1 and zones 3 than the losing team. Previous research results [33] that explicitly pointed to a very high rate of goal scoring in zone 1, where the present study was located (Fig 2). The author was surprised that no significant difference was found between the two sides in terms of shots in zone 2, but in zone 3 in contrast. Generally though shots from zone 2 are slightly further away from the goal compared to zone 3, but the angle of the shot is extremely conducive to scoring, while zone 3 is located on each side of the goal, with the perspective that the puck can be observed on the ice, hardly any angle of shot as long as the goalkeeper keeps a right position. In this regard, we suggest that it is the lack of defensive difficulty that makes the goalie tend to loosen up on zone 3, and stronger teams have seized upon this weak point, often cutting in at high speed or faking around the goal and then suddenly taking a quick released shot, which often serves as the game-winning goal in the unpredictable game.

Additionally, it is also something to do with both sides usually focusing heavily on defending in shooting zone 2, so the probability of blocking a shot in this area is higher, which produces more missed shots. Turning to zone 4, according to the results of the two tests, it can be seen that shots from shooting zone 4 are not conducive to offense, and generally shooting zone 4 is also mostly forced to take a shot, from the side of the attacking zone facing the opponents forced checking and then realize that the puck was insecure and had to reluctantly finish a shot. It is also possible that the lack of strength in the women’s shooting relates to the fact that it makes it easier for the goalie to defend, but from the perspective of the offense should still try to control the shots in shooting zone 4, prolonged possession waiting for teammates to receive would be a better choice.

As expected, shooting performance is the essential determinant of a match’s outcome, as the results show that the winning team has the better shooting ability and awareness of the shot, so the SOG plays an important role in the outcome of the game. Meanwhile, numerous examples have shown that having more shots than the opponent may also result in a loss, as it is the quality of the shots that matters [34,35], which is why SG and SOG are both important predictors of the game outcome.

Previous studies [36] have shown that in men’s international level games, about 30% of goals are scored through counterattacks, while in the Beijing Olympics women’s ice hockey scored 71.7% of goals through fast attacks, which can be interpreted from two perspectives, one of which may be attributed to the evolution of the sport and its development, which has made the feature of “fast” more and more pronounced. On the other hand, perhaps there is a huge difference between the men’s and women’s ice hockey games. Due to the fact that women’s ice hockey players are not allowed to engage in physical collisions, the team places greater emphasis on the technical and tactical aspects of the game, whereas men’s ice hockey focuses particularly on gaining an advantage in the area of body checking, which is mentioned by most scholars [37,38] as one of the main skills in ice hockey. However, it is an established trend that women’s ice hockey is getting closer to men’s hockey, and the future of the game will be more of a perfect blend of physical and technical skills.

Regarding the game period, another important indicator of fast performance that we have found to predict the outcome of games was the 2^nd^ period (OR = 1.541). This explains that the distance between the bench to the DZ increasing and to OZ decreasing had a significant influence on the offensive efficiency. In this context, more difficult and growing energy consumption of defender changes in the 2^nd^ period are undoubtedly the perfect timing for the offensive team to organise their attacks and therefore 2^nd^ period score at a higher rate. For this reason, one can always see that the team in possession of the puck takes advantage of the opponent to switch after a dump-in by quickly regrouping in the NZ, or by taking a long pass directly from the DZ to a forward close to the OZ blue line to launch a second attack. On this basis, it can be considered that it is necessary to maintain continuity in attack, and this involves the ability to regroup [39], which the winning team is aware of to achieve more odd-man rushes that lead to score at a high percentage. Meanwhile, we can also learn that it is essential to reduce dump-in during the 2^nd^ period, although this is difficult for the weaker teams.

Finally, we provide evidence that the winning team had more fast attacks in the PK situation, which implies that successful teams require a strong sense of counter-attacking and risk-taking to create opportunities and take advantage of them even with fewer players, while on the contrary, it also signals that numerical superiority does not conducive to increase the efficiency of fast attacks at all times and that the PP side needs to be equally defensive-minded.

A study conducted on men’s and women’s ice hockey revealed distinct differences in the characteristics of successful quick offense. In men’s hockey, success is strongly tied to tackling ability, intercepting the puck, executing one-timer shot techniques, performing well in specific shooting areas, and the second period of the game. Conversely, women’s teams place a greater emphasis on individual technique and shooting performance throughout the game. The effectiveness of fast attacks in women’s hockey is primarily associated with attacking efficiency and shot quality. At the same time, the fast offense in women’s hockey still focuses on steals and quick offense, which dovetails with some of the commonalities in the men’s game. In the men’s and women’s competitions, the second period of the game is considered to be the most noticeable phase of the rapid attack, which may be related to changes on the court, energy consumption and tactical adjustments. However, despite some commonalities, the differences in men’s and women’s hockey games are still noticeable. Men’s competitions may focus more on strength and speed, while women’s competitions may place more emphasis on technique and teamwork. These differences may be reflected in the strategy and execution of the quick offense, which presents unique challenges and opportunities for athletes of different genders. Therefore, an in-depth study of these differences and similarities in men’s and women’s hockey matches can contribute to a more complete understanding of the performance and tactical orientation of both genders in this field of the sport.

### 4.2 Limitations

Although this study provides valuable insights into the impact of fast attack tactics on the outcome of women’s ice hockey matches at the Beijing Winter Olympics, certain limitations and areas for improvement must be acknowledged:

The study predominantly centers on analyzing women’s ice hockey games during the Beijing Winter Olympics and therefore did not address potential changes that may have occurred in men’s ice hockey games. It is essential to acknowledge that certain games within the dataset exhibit notable score differentials, introducing a potential influence on the study’s outcomes. Notably, the examination in this study is confined to eight categories, such as ways of gaining possession, zone of gaining possession, and shot type. However, it is imperative to recognize that a fast attack encompasses numerous influencing factors, such as patterns of fast attack, which were not included in this study due to the difficulty of its quantification This unexplored aspect holds significant potential for future investigations, particularly in advancing the scientific understanding of hockey tactics.

Consequently, it is recommended that future research endeavors should strive to encompass a broader range of tournaments, including different levels of competition, and employ larger sample sizes to enhance the objectivity of the findings. More attention should be paid to games with large score differentials and games with closer scores. In addition, researchers need to fully explore the differences between men’s and women’s hockey. This will provide a valuable avenue for future scientific research in the field of ice hockey and contributing to a more comprehensive understanding of the sport.

## 5. Conclusion

To conclude, this study based on match statistic analysis allows us to understand how hockey teams can win games by improving fast attack performance. The current results show that shooting zone, shooting type, game period and shooting performance are the most important aspects of fast attack performance in determining game outcomes. It indicates that the winners have a more successful fast attack performance, which is mainly reflected in stealing more pucks, using more one-timers, creating more fast attack opportunities during the 2^nd^ period, taking more shots and having higher SG%. So that means the key to success in the game depends on playing aggressive defence, being initiative to steal the puck, timing the attack properly, shooting with as much quickness and suddenness as possible from a better position, and with good shot awareness as well as maintaining quality of the shot.

This study, despite its valuable insights, exhibits notable limitations. Firstly, the sample size is relatively small, potentially restricting the generalizability of findings. The methodology employed might be subject to constraints, and variations in data collection or analysis methods could impact the robustness of the results. Additionally, the absence of an exploration into external validity raises questions about the study’s applicability to diverse contexts or populations within women’s ice hockey. In future research, expanding the sample size and considering diverse methods may enhance the study’s reliability. Furthermore, addressing these limitations by conducting in-depth analyses of specific variables or scenarios could provide a more nuanced understanding of the observed phenomena. Overall, acknowledging and mitigating these limitations will contribute to the refinement and advancement of research in women’s ice hockey analytics. Future research in women’s ice hockey analytics could explore advanced statistical models to discern nuanced patterns. Investigating the impact of evolving game strategies, technological interventions, or rule changes on performance metrics would provide valuable insights. Additionally, exploring interdisciplinary collaborations with sports science and psychology could enrich the understanding of player behavior and decision-making. This avenue of research has the potential to uncover novel dimensions in women’s ice hockey analytics, contributing to both the scholarly discourse and practical applications within the sport. Overall, future work will be required to confirm the present findings with larger sample sizes and for teams and games of different levels.

## Supporting information

S1 Data Modeling and prediction of winning and losing rule under fast attack variables. (XLSX)
